# Redefining Access: Transition from Conventional to Transaxillary Endoscopic Aortic Valve and Ascending Aorta Replacement—A Case Report with Literature Review

**DOI:** 10.3390/jcm15155996

**Published:** 2026-08-01

**Authors:** Tanja Josic, Mirko Doss

**Affiliations:** Department of Cardiac Surgery, Helios Hospital Siegburg, 53721 Siegburg, Germany; tanja.josic@helios-gesundheit.de

**Keywords:** minimally invasive cardiac surgery, transaxillary approach, bicuspid aortic valve, ascending aortic aneurysm, aortic valve replacement, RAM system, first-in-human

## Abstract

**Background:** Minimally invasive approaches for combined aortic valve and ascending aortic surgery remain challenging. We report a case of utilizing a transaxillary approach (TAX) in combination with the RAM^®^ system. **Methods:** A 65-year-old male presented with exertional angina and palpitations. He reported a one-year history of dizziness. Diagnostics revealed severe aortic regurgitation due to a calcified bicuspid aortic valve and an ascending aortic aneurysm measuring 57 × 54 mm. Left ventricular ejection fraction was reduced to 42%. Coronary artery disease was excluded. Surgery was performed via a right transaxillary mini-thoracotomy (3rd intercostal space) using endoscopic visualization. Cardiopulmonary bypass was established through femoral cannulation. After aortic cross-clamping and cardioplegic arrest, the bicuspid valve was excised and replaced with a bioprosthesis. A supracoronary ascending aortic replacement was performed using a Dacron graft. The RAM^®^ system was used for annular suturing and proximal anastomosis, with automated fastener fixation. **Results:** The patient was extubated on postoperative day 1 and transferred to intermediate care on day 2. Postoperative recovery was uneventful, with no neurological deficits, bleeding, or other complications. Discharge occurred on postoperative day 9 in stable condition. **Conclusions:** This case highlights the feasibility and safety of a minimally invasive transaxillary approach for combined aortic valve and ascending aortic replacement using the RAM^®^ system. This technique may expand the surgical armamentarium for complex aortic pathology while avoiding sternotomy. Further studies are required to evaluate reproducibility, long-term outcomes, and broader applicability.

## 1. Background

Minimally invasive cardiac surgery has become an established alternative to conventional median sternotomy for a variety of cardiac procedures [1,2]. Continuous advances in imaging, endoscopic visualization, peripheral cannulation techniques, specialized instrumentation, and automated suturing technologies have enabled increasingly complex operations to be performed through limited-access approaches while maintaining the principles of conventional cardiac surgery [3,4,5,6].

Among these techniques, the transaxillary (TAX) approach has recently emerged as an attractive access route for minimally invasive aortic valve surgery. By completely preserving the sternum, it offers excellent cosmetic results, reduced surgical trauma, preservation of thoracic stability, and favorable exposure of the aortic root and ascending aorta. Recent series from experienced centers have demonstrated excellent perioperative outcomes for isolated aortic valve replacement using this approach [2,7,8].

Despite these advances, in patients with combined pathology of the aortic valve and ascending aorta—particularly in the setting of bicuspid aortic valve (BAV)—standard treatment remains median sternotomy because of technical complexity and limited operative exposure.

Nevertheless, reports regarding combined replacement of the aortic valve and ascending aorta through a transaxillary endoscopic approach remain extremely limited. Furthermore, reproducible methods facilitating secure suturing and prosthesis implantation in such restricted operative fields are still evolving. The limited operative space associated with minimally invasive approaches creates significant technical challenges, particularly during annular suturing, prosthesis implantation and construction of aortic anastomoses [2].

The RAM^®^ system (LSI Solutions Inc., Victor, NY, USA) is an automated double-curved needle suturing device developed to facilitate reproducible placement of pledgeted mattress sutures during minimally invasive cardiac surgery [9].

We report the first-in-human case of an endoscopic transaxillary combined aortic valve replacement and supracoronary ascending aortic replacement using the RAM^®^ system.

## 2. Case Presentation

A 65-year-old male patient presented to the cardiology department with suspected coronary artery disease due to exertional angina pectoris and frequent ventricular extrasystoles. Until this presentation, the patient had never undergone previous cardiological evaluation and had not been receiving any chronic medication. He reported a one-year history of dizziness, previously evaluated neurologically without identification of a causative pathology. During the weeks preceding admission, he developed intermittent chest pain and palpitations. Electrocardiography demonstrated ventricular triplets, and long-term rhythm monitoring was initiated.

Transthoracic echocardiography revealed severe aortic regurgitation caused by a heavily calcified bicuspid aortic valve. Left ventricular function was already impaired, with a left ventricular ejection fraction (LVEF) of 42%. Coronary angiography excluded significant coronary artery disease.

Computed tomography (CT) imaging confirmed an aneurysmal dilation of the ascending aorta measuring 57 × 54 mm, with otherwise normal aortic dimensions and no significant atherosclerotic burden. The aortic valve morphology corresponded to a bicuspid aortic valve type I with fusion of the right and left coronary cusps (Sievers classification for BAV) [10]. Extensive calcification involving the valve leaflets and the aorto-mitral continuity was observed. No relevant atherosclerotic disease of the thoracic or abdominal aorta was present. No pulmonary embolism, infectious focus, or malignancy was detected.

In our institution, the EuroSCORE II is routinely calculated for every patient during the preoperative assessment. For the present patient, the EuroSCORE II was 1.4%, indicating a low predicted operative risk [11].

Based on the combined pathology consisting of severe aortic insufficiency and ascending aortic aneurysm, surgical treatment was indicated.

### 2.1. Preoperative Evaluation and Patient Selection

Appropriate patient selection is essential for the safe and successful performance of transaxillary minimally invasive aortic surgery. At our institution, all candidates undergo a standardized preoperative evaluation consisting of comprehensive clinical assessment, multimodality imaging, and functional testing to determine their anatomical suitability for this approach.

The routine preoperative workup includes electrocardiography, transthoracic and/or transesophageal echocardiography, coronary angiography, laboratory testing and contrast-enhanced computed tomography of the thoracic aorta. CT imaging represents the cornerstone of our preoperative planning, as it allows detailed assessment of both the underlying pathology and the feasibility of the transaxillary access route.

Besides evaluating the extent of aortic disease, CT is systematically analyzed to determine the spatial relationship between the ascending aorta, aortic root and anterior chest wall. Particular attention is paid to the orientation of the ascending aorta within the thoracic cavity, the anticipated surgical working angle, the position of the aortic root relative to the third intercostal space and the expected instrument reach.

**Figure 1:**
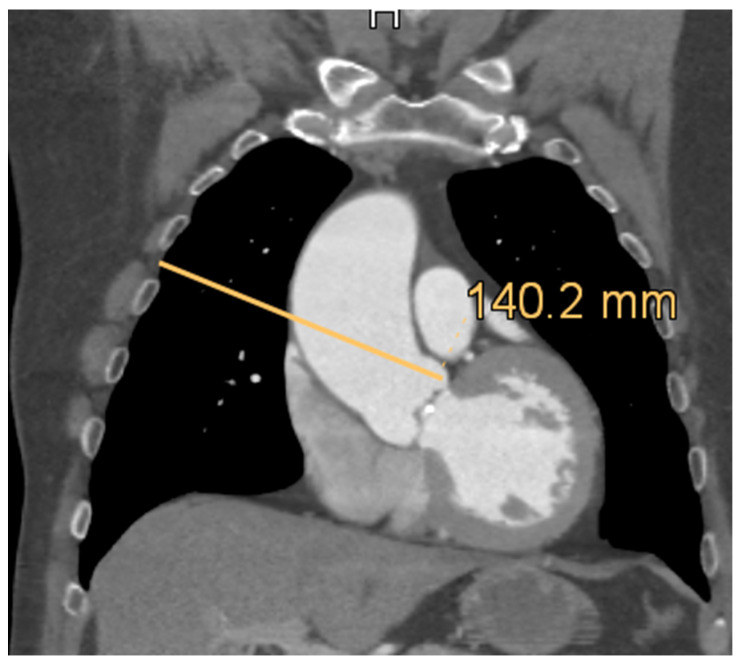
Preoperative coronal CT scan illustrating the institutional measurement used for patient selection for the transaxillary minimally invasive approach. The distance is measured (yellow line) from the level of the third intercostal space, corresponding to the planned surgical access, to the aortic root along the anticipated operative trajectory. In the present case, the measured distance was 14 cm, fulfilling the anatomical selection criteria.

As part of our institutional selection algorithm, the distance between the aortic root and the level of the third intercostal space is routinely measured on preoperative CT scans. The CT-based measurement used for patient selection is illustrated in Figure 1.

This measurement is performed along the anticipated operative trajectory of the transaxillary approach and serves as one of the key anatomical criteria for patient selection. Based on our institutional experience, a distance of ≤17 cm provides optimal exposure, sufficient instrument reach, and favorable operative angles, thereby facilitating safe manipulation during minimally invasive surgery. Patients exceeding this threshold are carefully evaluated individually, as greater distances may substantially compromise visualization and instrument maneuverability.

Peripheral vascular anatomy is evaluated to determine the feasibility of femoral arterial and venous cannulation for cardiopulmonary bypass.

**Figure 2:**
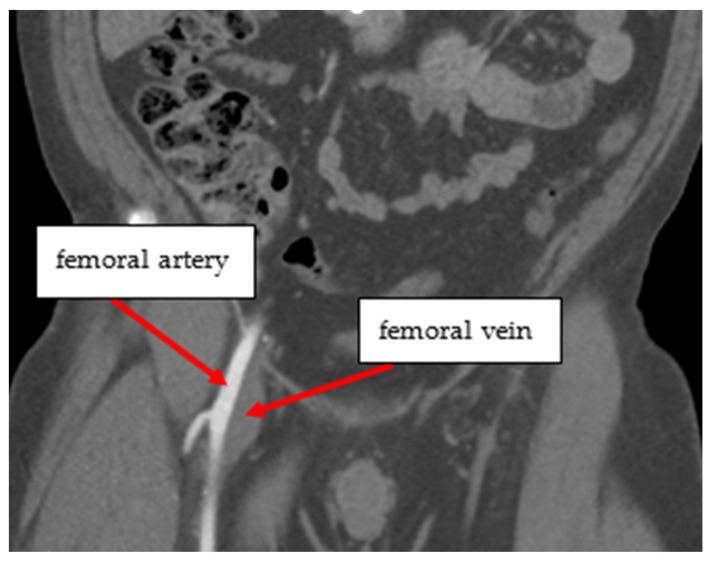
Coronal CT scan of the groin illustrating the assessment of the femoral vessels. The red arrow marks the common femoral artery and common femoral vein intended for peripheral cannulation.

As shown in Figure 2, preoperative contrast-enhanced computed tomography depicts the femoral vessels used for peripheral cannulation. The red arrow indicates the common femoral artery and common femoral vein, which were selected for femoral cannulation. In the present case, the femoral artery showed no relevant calcification or significant atherosclerotic disease, making it suitable for safe arterial cannulation during cardiopulmonary bypass.

Further selection criteria include previous thoracic surgery, severe chest wall deformities, extensive aortic calcification (porcelain aorta), significant peripheral vascular disease and other patient-specific anatomical factors that may limit safe transaxillary access. Patients presenting with these findings are generally considered unsuitable for this minimally invasive approach.

The patient presented in this report fulfilled all anatomical and clinical selection criteria, including the CT-based measurements illustrated in Figure 1, and was therefore considered an appropriate candidate for minimally invasive transaxillary replacement of the aortic valve and ascending aorta.

After detailed preoperative counseling regarding the advantages and disadvantages of mechanical and biological prostheses, the 65-year-old patient explicitly opted for a biological valve to avoid lifelong anticoagulation with a vitamin K antagonist. An INSPIRIS RESILIA bioprosthesis (Edwards Lifesciences, Irvine, CA, USA) was selected according to our institutional practice and the encouraging long-term durability data reported for RESILIA tissue technology [12,13].

### 2.2. The RAM^®^ System—Surgical Device

**Figure 3:**
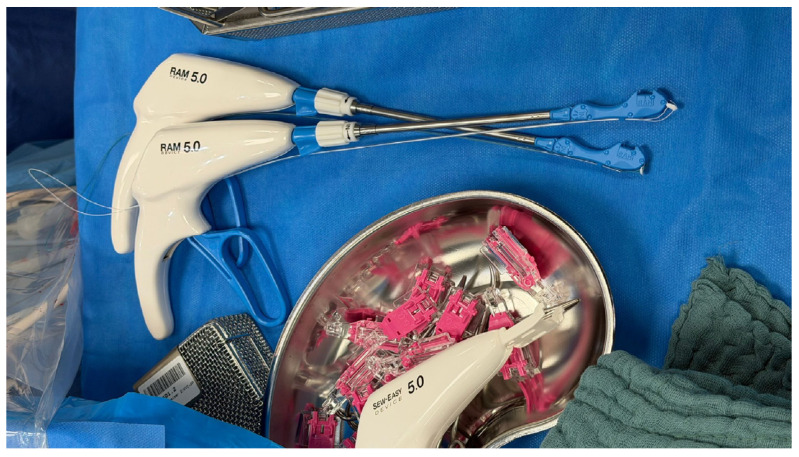
The RAM^®^ system, an automated curved double-needle suturing device.

As shown in Figure 3, the RAM^®^ system is an automated curved double-needle suturing device specifically developed for minimally invasive cardiac surgery. The RAM^®^ device is available in two configurations, providing either a 3.5-mm or a 5-mm spacing between the two curved needles, allowing the surgeon to select the appropriate needle distance according to the surgical application and tissue characteristics [9].

The concept of automated double-needle suturing was first introduced into clinical practice in 2017, when the technology was successfully applied for automated epicardial pacemaker lead fixation and subsequently described in selected cases of minimally invasive mitral valve surgery. These early experiences demonstrated the feasibility of standardized automated suture placement in confined operative fields and laid the foundation for further development of the technology [14,15].

The system was designed to facilitate standardized and reproducible placement of pledgeted mattress sutures within restricted operative fields. By simultaneously advancing two curved needles through the target tissue in a predefined trajectory, the device improves surgical ergonomics, reduces manual needle manipulation, improving suture consistency and facilitating precise suture placement in anatomically challenging locations [9].

Initial clinical experience with the RAM^®^ system has primarily been reported in minimally invasive totally endoscopic aortic and mitral valve replacement, where it has been shown to facilitate annular suture placement while maintaining procedural safety and reproducibility [16]. Despite its increasing adoption in selected centers, published experience remains limited and has almost exclusively focused on valve implantation [3,16,17].

To the best of our knowledge, the use of the RAM^®^ system for construction of the ascending aortic graft anastomosis has not previously been described.

In the present case, we therefore expanded the application of the device beyond its established indication by combining it with guided prosthesis deployment during minimally invasive transaxillary replacement of the ascending aorta.

Combined with automated fastener systems such as Cor-Knot^®^ (LSI Solutions Inc., Victor, NY, USA) [18], the RAM^®^ system may simplify annular and aortic anastomoses and potentially expand the applicability of minimally invasive surgery to more complex aortic pathologies.

### 2.3. Surgical Technique

Following induction of general anesthesia, transesophageal echocardiography was performed intraoperatively. The patient was positioned supine with slight elevation of the right hemithorax. Cardiopulmonary bypass was established via percutaneous cannulation of the right femoral artery and vein under echocardiographic guidance after systemic heparinization.

A right transaxillary mini-thoracotomy measuring approximately 4 cm was created through the third intercostal space. A soft tissue retractor was inserted, followed by placement of a 30-degree endoscopic camera. Continuous carbon dioxide insufflation was initiated within the operative field.

After opening the pericardium anterior to the phrenic nerve, multiple pericardial stay sutures were placed to optimize exposure. Venting of the left ventricle was achieved via the right superior pulmonary vein through a separate incision.

A Chitwood clamp was introduced through a separate stab incision and positioned around the ascending aorta. Following aortic cross-clamping, cardioplegic arrest was achieved using crystalloid Bretschneider (Custodiol^®^—Registered Trademark of Dr. Franz Köhler Chemie GmbH, Bensheim, Germany) cardioplegia solution administered directly into the coronary ostia.

**Figure 4:**
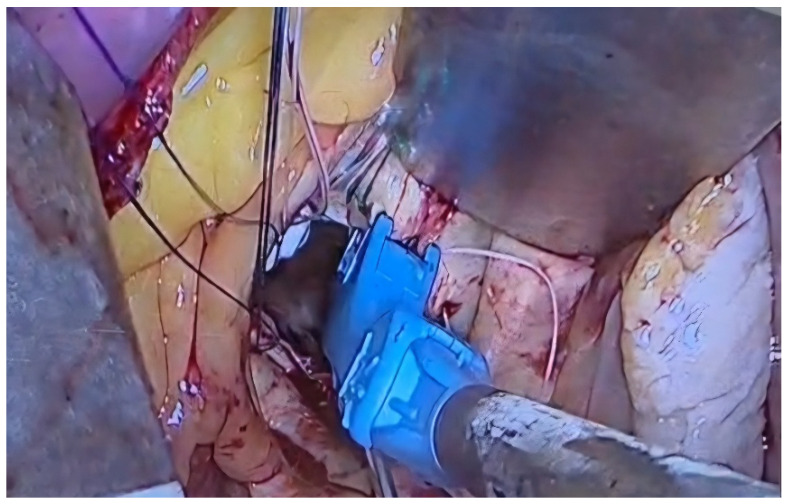
Implantation of the pledgeted 2-0 U-sutures placed subannularly with the RAM^®^ system.

Inspection of the aortic valve confirmed a congenital bicuspid morphology with fusion of the right and left coronary cusps. Significant calcification extended into the aorto-mitral continuity. Following complete decalcification of the native bicuspid aortic valve and the aorto-mitral continuity, pledgeted mattress annular sutures were circumferentially placed using the RAM^®^ system, as shown in Figure 4. The device enabled reproducible deployment of double-needle sutures despite the restricted endoscopic transaxillary working space.

After completion of annular suture placement, the sutures were passed through the sewing ring of the INSPIRIS RESILIA aortic valve bioprosthesis. The prosthesis was subsequently lowered into the annulus along the prepositioned sutures in a controlled manner. Knot fixation was performed using the Cor-Knot^®^ automated fastening system.

Subsequently, supracoronary replacement of the ascending aorta was performed using a Dacron graft. In contrast to the conventional technique of constructing the proximal graft anastomosis with a continuous running suture, our institutional approach utilizes the RAM^®^ system to facilitate precise and reproducible graft implantation.

**Figure 5:**
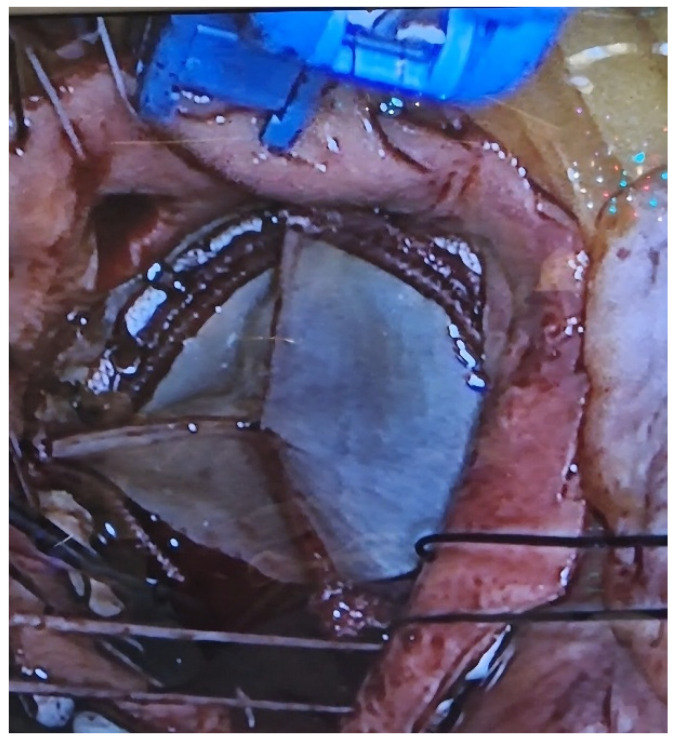
INSPIRIS RESILIA bioprosthetic valve in position; proximal aortic stump anastomosis was performed using the RAM^®^ system.

After preparation of the proximal aortic stump, 17 evenly distributed 5-mm pledgeted mattress sutures were placed circumferentially using the RAM^®^ device, as shown in Figure 5. After passage of the sutures through the sewing cuff of the Dacron graft, the graft was advanced into its final position by simultaneously sliding it along the pre-positioned sutures. This “parachuting” maneuver enables controlled, uniform, and precise positioning of the graft, particularly within the limited operative field of the transaxillary approach. The sutures were subsequently secured using Cor-Knot^®^ fasteners, resulting in rapid completion of the proximal anastomosis.

**Figure 6:**
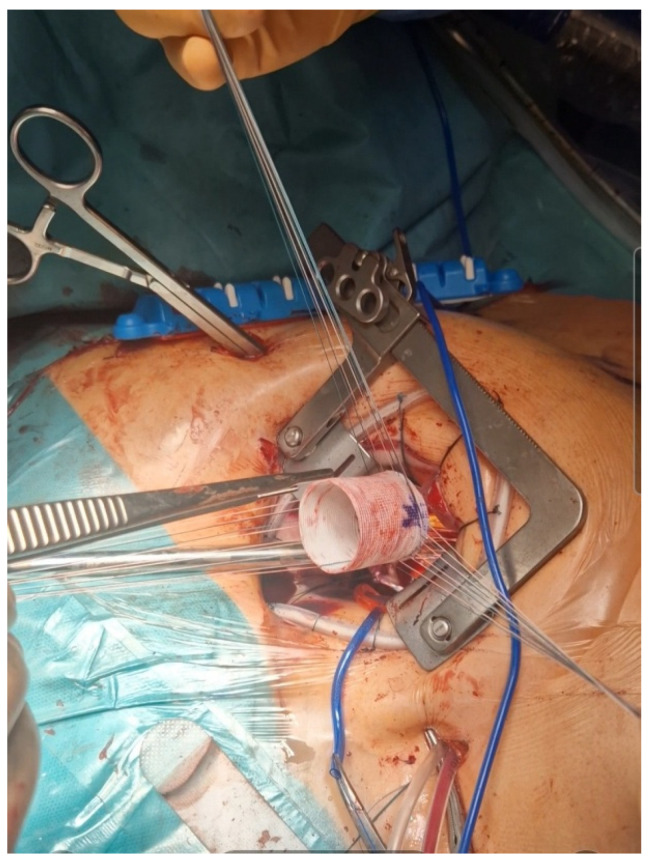
Josic technique, facilitating smooth graft positioning along prepositioned sutures.

**Figure 7:**
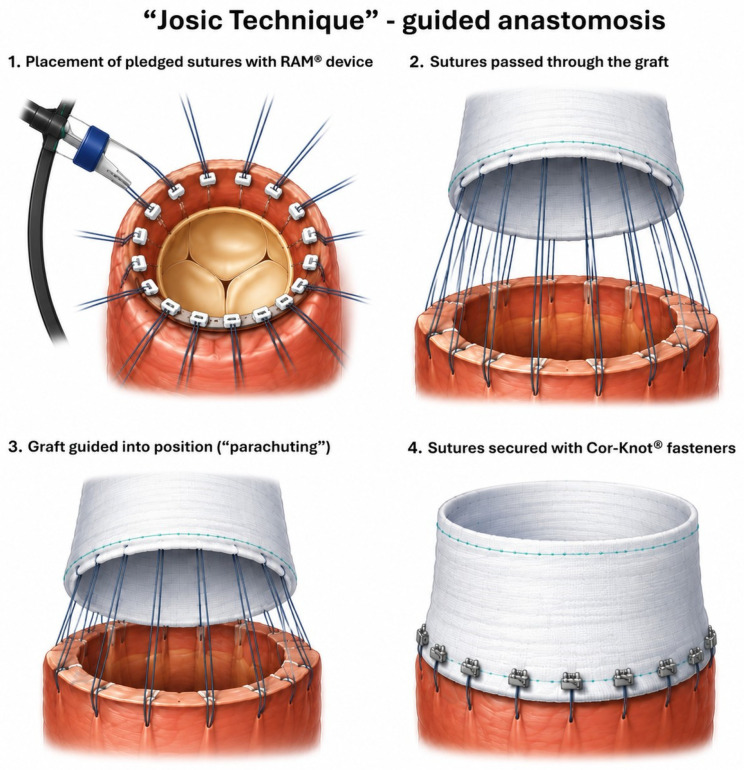
Step-by-step illustration of the “Josic technique” for ascending aortic graft implantation.

Within our institution, this guided graft deployment strategy has been informally termed the “Josic technique” (Figure 6). The term is used solely as an institutional designation for the technical concept described in this report and should be regarded as a proof-of-concept rather than an established surgical technique. Further clinical experience is required to evaluate its reproducibility and potential clinical benefit. The principle of the guided graft deployment (Josic technique) is illustrated step-by-step in Figure 7.

The technique combines circumferential pre-placement of multiple pledgeted sutures with guided prosthesis deployment, thereby simplifying graft implantation and improving handling in minimally invasive surgery.

In this case, the RAM^®^ system was intentionally applied only to the proximal anastomosis as an initial proof-of-concept evaluation of the technique. The distal ascending aortic anastomosis was therefore performed according to the standard institutional technique using a continuous 4-0 polypropylene suture (PROLENE® (Ethicon, Raritan, NJ, USA).

Following careful de-airing, the aortic clamp was removed. The heart resumed spontaneous sinus rhythm with satisfactory myocardial recovery. Temporary epicardial pacing wires were placed on the right atrium and ventricle.

After adequate reperfusion, cardiopulmonary bypass was discontinued under low-dose inotropic support. Transesophageal echocardiography demonstrated normal prosthetic valve function without paravalvular leakage and satisfactory graft position.

The femoral vessels were decannulated and vascular closure was achieved using a Perclose ProGlide™ Suture-Mediated Closure System (Abbott Vascular, Redwood City, CA, USA) [19]. Hemostasis was achieved and the procedure concluded with placement of a right pleural drain and layered wound closure.

Total operative time was 350 min, cardiopulmonary bypass time was 237 min, and aortic cross-clamp time was 128 min.

## 3. Results

### 3.1. Perioperative Results

The procedure was completed successfully without conversion to sternotomy. Intraoperative hemodynamics remained stable with spontaneous recovery of sinus rhythm after reperfusion.

### 3.2. Postoperative Intesive Care Unit

Postoperatively, blood pressure was strictly controlled below 100 mm of mercury (mmHg) during the first 24 h to minimize stress on the proximal and distal aortic reconstruction.

The patient was extubated on postoperative day one and transferred to the intermediate care unit on postoperative day two.

### 3.3. Complications

A transient rapid atrial fibrillation occurred postoperatively without hemodynamic compromise and was managed conservatively.

No neurological deficits, bleeding complications, wound complications, or respiratory insufficiency occurred.

### 3.4. Postoperative Echocardiographic Findings

Postoperative transthoracic echocardiography demonstrated normal cardiac chamber dimensions and a well-positioned 30-mm supracoronary ascending aortic prosthesis. Left ventricular systolic function was mildly to moderately reduced, with a LVEF of 46%. No additional regional wall motion abnormalities were observed. Diastolic function was consistent with grade II diastolic dysfunction with elevated left ventricular filling pressures.

Right ventricular systolic function was mildly reduced (Tricuspid Annular Plane Systolic Excursion —TAPSE 1.8 cm).

The implanted 25-mm INSPIRIS RESILIA bioprosthetic aortic valve demonstrated normal function without structural dysfunction or paravalvular regurgitation. Doppler assessment showed a peak transvalvular gradient of 25 mmHg, a mean gradient of 13 mmHg, and a peak velocity of 2.4 m/s. The calculated effective orifice area was 2.0 cm^2^, indicating satisfactory prosthetic valve performance.

No pericardial effusion was detected. A small left-sided pleural effusion (<150 mL) was present, whereas no right-sided pleural effusion was observed.

### 3.5. Postoperative Antithrombotic Therapy

Postoperatively, the patient received standard antithrombotic therapy with low-dose aspirin, in accordance with the current ESC/EACTS guidelines for surgical biological aortic valve replacement, as no additional indication for oral anticoagulation was present [20].

**Figure 8:**
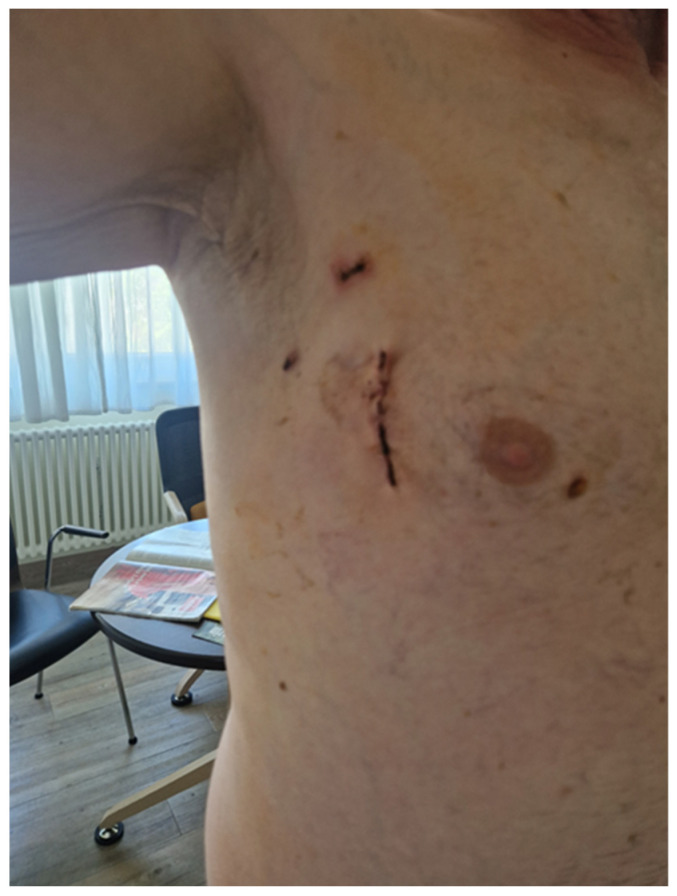
Postoperative view of the TAX access with the cosmetic result.

The patient was discharged home on postoperative day nine in stable condition with good functional recovery. The postoperative cosmetic result is shown in Figure 8.

## 4. Discussion

Minimally invasive cardiac surgery continues to evolve toward increasingly complex procedures performed through progressively smaller access routes. While minimally invasive isolated aortic valve replacement has become well established in experienced centers, combined procedures involving the ascending aorta remain technically demanding because of restricted visualization, limited maneuverability, and complexity of anastomotic reconstruction [1,2,4,6,21,22,23,24].

While transaxillary minimally invasive aortic valve replacement has shown promising outcomes in large contemporary series, combined procedures involving the ascending aorta are still rarely reported. Although it offers several well-recognized advantages, including complete sternal preservation, excellent cosmetic results, favorable exposure of the aortic root and rapid postoperative recovery, it is not without limitations [2,7,8].

The largest published series to date by Wilbring et al., including more than 1000 consecutive transaxillary aortic valve procedures, demonstrated that the transaxillary approach provides excellent perioperative outcomes comparable to conventional median sternotomy while maintaining the well-recognized benefits of minimally invasive surgery. However, the authors also emphasized that this technique should currently be performed in specialized high-volume centers with dedicated expertise in minimally invasive cardiac surgery [8].

Despite these encouraging results, several authors have highlighted important limitations of the transaxillary approach. In a subsequent study evaluating the safety of transaxillary access, Wilbring et al. reported an increased rate of re-exploration for bleeding, predominantly related to intercostal vessel injury or muscular bleeding associated with the lateral thoracic access. Although these complications were manageable, they underline the importance of meticulous surgical technique and institutional experience [7].

Similarly, Kowalówka et al. emphasized that successful implementation of the transaxillary approach depends on careful CT-based anatomical patient selection and should be restricted to experienced high-volume centers. The authors further described the potential for vascular access-related complications associated with femoral cannulation as well as an increased incidence of perioperative stroke, which may be related to guidewire manipulation during femoral cannulation and/or retrograde arterial perfusion. Importantly, they also demonstrated a substantial institutional learning curve, estimating that approximately 30–50 procedures are required to achieve procedural proficiency, after which operative, cardiopulmonary bypass, and aortic cross-clamp times decrease significantly [2].

Taken together, the available evidence suggests that the transaxillary approach is a safe and effective minimally invasive alternative in carefully selected patients when performed in experienced centers. Nevertheless, careful patient selection, standardized operative strategies and progressive institutional experience remain essential prerequisites for minimizing complications and optimizing clinical outcomes. The observed reduction in operative, cardiopulmonary bypass and aortic cross-clamp times with increasing experience further emphasizes the importance of the learning curve when implementing this advanced minimally invasive technique [2,7,8,25].

Restricted visualization, limited maneuverability, and constrained instrument angulation become particularly problematic during annular suturing and aortic reconstruction. Consequently, reproducible implantation techniques are essential.

One of the most innovative aspects of the present case is the expanded application of the RAM^®^ system beyond isolated valve implantation.

The currently available clinical experience with the RAM^®^ system remains limited. The first published clinical series by El-Sayed et al. demonstrated the feasibility and safety of automated annular suturing during totally endoscopic aortic and mitral valve replacement, reporting successful implementation of the technology in routine minimally invasive practice [16].

Subsequent propensity score-matched analysis by Salamate et al. showed that automated annular suturing using the RAM^®^ system achieved surgical outcomes comparable to conventional hand-sewn techniques while significantly reducing aortic cross-clamp time and facilitating reproducible annular suture placement [3]. These findings suggest that automated suturing technology may improve procedural efficiency without compromising surgical safety.

To date, the published literature has focused almost exclusively on annular suture placement during minimally invasive valve implantation. Application of the RAM^®^ system for vascular reconstruction, particularly for construction of the proximal ascending aortic graft anastomosis, has not previously been described.

In the present case, we expanded the application of the RAM^®^ system beyond its established indication. The RAM^®^ system facilitated standardized and reproducible placement of pledgeted-supported sutures at both the annular level and the proximal ascending aorta. This substantially improved procedural ergonomics within the confined operative field.

Combined with Cor-Knot^®^ fasteners, which have previously been associated with reduced technical complexity and enhanced efficiency in minimally invasive valve surgery, this approach allowed controlled deployment of both the valve prosthesis and vascular graft [5].

One particularly relevant technical aspect was the guided lowering of the prosthesis along prepositioned pledgeted sutures. This strategy significantly improved prosthesis control and orientation during implantation. Especially the distal part of the anastomosis underneath the closed sternum, facing away from the surgeon, can be challenging to perform conventional suturing.

Through repeated procedural optimization, this technique evolved into a reproducible workflow within our institution.

Although operative and cardiopulmonary bypass times were longer than those generally reported for isolated minimally invasive aortic valve replacement, this likely reflects the complexity of the present procedure, involving combined aortic valve and ascending aortic replacement through an endoscopic transaxillary approach, as well as the early learning curve associated with implementation of a novel suturing technology. Previous studies investigating minimally invasive transaxillary aortic valve surgery demonstrated significant reductions in operative times with increasing institutional experience in transaxillary cardiac surgery [8].

Similar observations have been reported by Salamate et al., who evaluated the implementation of the RAM^®^ system for endoscopic isolated aortic valve replacement. During the first year following introduction of the technology, the authors reported median cardiopulmonary bypass time of approximately 160 min and median cross-clamp time per year over 80 min, despite the less complex nature of isolated valve replacement. With increasing surgical experience, operative efficiency improved substantially, resulting in a progressive year-by-year reduction in the median cardiopulmonary bypass and aortic cross-clamp time [3]. These findings highlight the importance of the institutional learning curve when introducing novel technologies into minimally invasive cardiac surgery.

Importantly, despite the technical complexity and prolonged operative duration, the patient experienced rapid postoperative recovery without major complications. Preservation of thoracic stability through avoidance of sternotomy may contribute to accelerated mobilization, reduced postoperative discomfort, and improved respiratory recovery.

## 5. Limitations

Several limitations of the present report must be acknowledged.

First, this study describes a single first-in-human case performed in a highly selected patient within an experienced minimally invasive cardiac surgery center. Therefore, the reproducibility and generalizability of the described technique remain uncertain.

Second, patient selection represents a critical factor for successful transaxillary combined aortic surgery. Favorable anatomical conditions, including adequate spatial relationship between the ascending aorta and thoracic wall, absence of severe peripheral vascular disease and limited aortic calcification, were essential prerequisites in the present case. Consequently, this approach may not be applicable to all patients requiring complex aortic surgery.

Third, operative, cardiopulmonary bypass, and cross-clamp time remained prolonged compared to conventional sternotomy approaches. Although this likely reflects the initial learning curve associated with a novel minimally invasive technique, the impact of extended operative duration on broader patient populations remains unknown.

Furthermore, no long-term follow-up data are currently available regarding durability of the anastomosis constructed using the RAM^®^ system. While early postoperative imaging demonstrated excellent graft and valve function, long-term stability and clinical outcomes require further investigation.

Another important limitation concerns the learning curve associated with both the transaxillary approach and the use of automated suturing technologies. Successful implementation requires extensive experience in minimally invasive cardiac surgery, peripheral cannulation strategies, endoscopic visualization and complex aortic procedures. Therefore, transferability to lower-volume centers may initially be limited.

In addition, although the RAM^®^ system appeared to improve ergonomics and reproducibility within the restricted operative field, no comparative data currently exist demonstrating superiority over conventional hand-sewn techniques. The potential advantages regarding procedural efficiency, safety, or postoperative recovery remain speculative and require prospective evaluation.

Future studies involving larger patient cohorts are necessary to evaluate clinical outcomes, reproducibility, learning curves, and long-term durability. Larger prospective studies and multicenter experiences are necessary to determine whether the described approach provides measurable benefits compared to established minimally invasive or conventional surgical techniques. Additionally, further refinement of dedicated minimally invasive instruments and automated suturing technologies may continue expanding the role of transaxillary cardiac surgery.

## 6. Conclusions

This first-in-human case highlights the technical feasibility and early safety of minimally invasive transaxillary combined aortic valve and ascending aortic replacement, using the RAM^®^ system.

The described approach combines sternotomy avoidance with reproducible annular and aortic reconstruction through a highly limited operative field. Automated suturing and fastening technologies may play an important role in expanding minimally invasive strategies toward increasingly complex aortic procedures.

Further clinical experience and larger studies are required to evaluate reproducibility, long-term durability, and broader applicability of this novel technique.

## Data Availability

The original contributions presented in this study are included in the article. Further inquiries can be directed to the corresponding author.

## Abbreviations

BAV	bicuspid aortic valve
CA	California
CT	Computed tomography
LVEF	left ventricular ejection fraction
MICS	Minimally invasive cardiac surgery
mmHg	millimeters of mercury
NJ	New Jersey
NY	New York
TAPSE	Tricuspid Annular Plane Systolic Excursion
TAX	transaxillary approach
USA	United States of America
